# Improving Efficiency and Communication around Sedated Fracture Reductions in a Pediatric Emergency Department

**DOI:** 10.1097/pq9.0000000000000135

**Published:** 2019-02-13

**Authors:** Niloufar Paydar-Darian, Michael P. Goldman, Kenneth A. Michelson, Katharine C. Button, Elizabeth K. Hewett, Theodore E. Macnow, Andrew F. Miller, Megan A. Musisca, Joel D. Hudgins, Matthew A. Eisenberg

**Affiliations:** From the * Division of Emergency Medicine, Boston Children’s Hospital and Harvard Medical School, Boston, Mass.; † Division of Pediatric Emergency Medicine, Yale University School of Medicine, New Haven, Conn.; ‡ Division of Emergency Medicine, Children’s National Medical Center, Wash.; § Division of Pediatric Emergency Medicine, Children’s Hospital of Pittsburgh of UPMC, Pittsburgh, Pa.; ¶ Division of Pediatric Emergency Medicine, University of Massachusetts Memorial Children’s Medical Center, Worcester, Mass.

## Abstract

**Introduction:**

Procedural sedation for fracture reduction in the pediatric emergency department (ED) is a time-consuming process requiring multidisciplinary coordination. We implemented a quality improvement initiative aimed at (1) decreasing mean ED length of stay (LOS) for children with sedated long bone fracture reductions by 15% over 12 months and (2) improving interdisciplinary communication around procedural sedation.

**Methods:**

Pediatric emergency medicine fellows at a children’s hospital designed and implemented an initiative targeting the efficiency of the sedation process. Interventions included a centralized sedation tracking board, a team member responsibility checklist, family handouts, early discharge initiatives, and postsedation review forms. We tracked progress via statistical process control charts and interdisciplinary communication by intermittent surveys.

**Results:**

Pediatric emergency medicine fellows performed 2,246 sedations during the study period. Mean LOS decreased from 361 to 340 minutes (5.8%) after implementation and demonstrated sustainability over the postintervention period. One hundred eight providers completed the preimplementation communication survey, with 58 and 64 completing surveys at 4 and 9 months postimplementation, respectively. The proportion reporting somewhat or strong satisfaction with communication increased from 68% at baseline to 86% at 4 months (*P* = 0.02) and 92% at 9 months (*P* < 0.001 versus baseline).

**Conclusions:**

A quality improvement initiative created a sustainable process to reduce ED LOS for sedated reductions while improving satisfaction with interdisciplinary communication.

## Abstract 2

Supplemental Digital Content is available in the text.

## INTRODUCTION

Long bone fractures are common among children presenting to an emergency department (ED).^1–3^ Management of long bone fractures often requires reduction under procedural sedation in the ED.^4–10^ However, sedated fracture reductions are resource intensive and require assembly of an interdisciplinary team, which can adversely affect ED efficiency.^11,12^

Previous reports have focused on the safety of procedural sedation in children, but few studies have targeted improvements in the efficiency of this process.^11–13^ Targeting efficiency is important, as improvements in ED efficiency and patient length of stay (LOS) are correlated with improved patient satisfaction.^14–18^ As a result, several studies have focused on specific interventions such as “front-end” operations improvements, among others, to streamline and improve ED throughput.^19,20^

Also, stakeholder focus groups and anecdotal reports both from our and other similar institutions indicated that interdisciplinary communication around such sedations is often lacking. This deficiency is due to a variety of factors, including the involvement of multiple services, the presence of rotating trainees who are unfamiliar with the standard processes, the providers’ patient load, and the lack of centralized communication between physician and nursing providers.

Thus, we conducted a quality improvement (QI) initiative targeting efficiency of procedural sedation for children undergoing long bone fracture reduction in our ED.

Our primary aim was to decrease the mean ED LOS for children undergoing ketamine sedation for long bone fractures by 15% over 12 months. Our secondary objective was to improve interdisciplinary communication around procedural sedation.

## METHODS

The Department of Medicine Performance Excellence Group, an internal committee that reviews QI initiatives, deemed this project QI and therefore did not require review and approval by the institutional review board.

### Setting

We conducted this QI initiative in the ED of a freestanding quaternary care children’s hospital, with approximately 60,000 visits and roughly 600 sedated fracture reductions annually.

The project involved the collaboration of 2 consecutive Accreditation Council for Graduate Medical Education-accredited fellowship classes of pediatric emergency medicine fellows.^21^ The fellows conceived, planned, and executed the project with faculty mentorship. Two project leaders were designated, and the remaining fellows were divided into a measurement team and an implementation team.

### Context

**Fig. 1.**
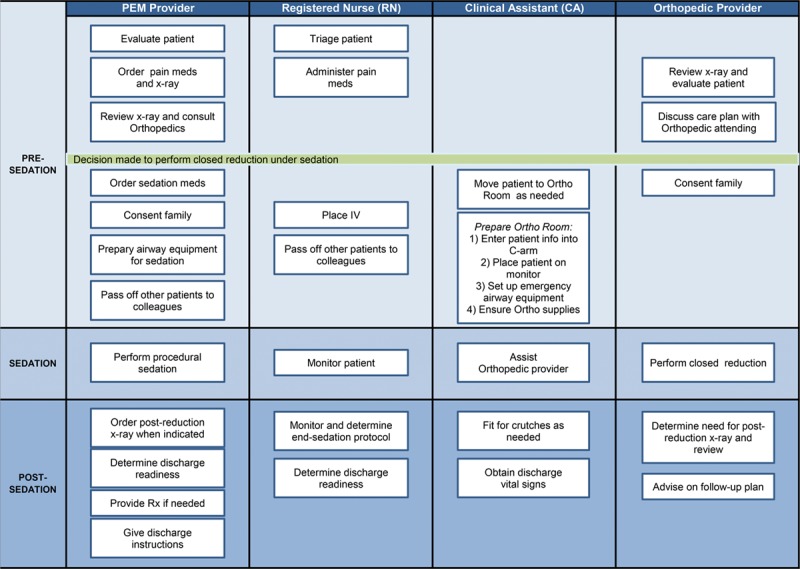
Flowchart of sedated fracture reductions.

The current process for sedated fracture reductions is described in Figure 1.

### Interventions

**Fig. 2.**
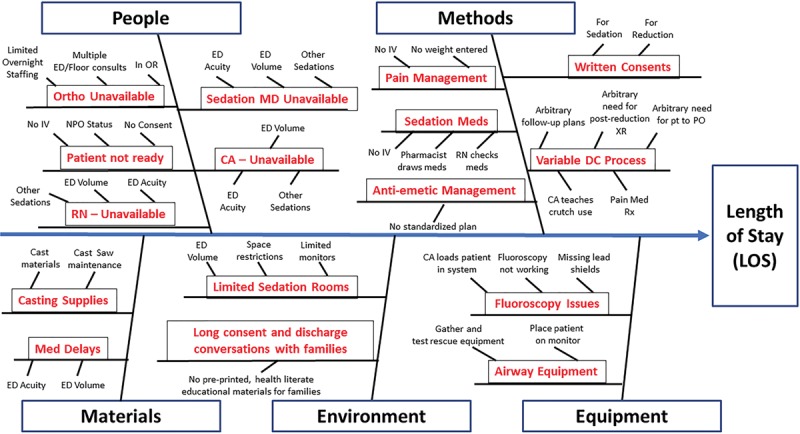
Cause-and-effect diagram addressing emergency department length of stay for those awaiting procedural sedation for long bone reduction.

The initiative began with a series of meetings of representatives from key stakeholders, including clinical assistants, nurses, and physicians from the ED and Department of Orthopedic Surgery. Together, a cause-and-effect diagram was generated (Fig. 2). We identified the following drivers as most influential on the target population’s LOS: assigned tasks for each team member, ED team member availability, the orthopedist’s overall caseload, communication among sedating team members, patient factors (eg, last meal time), and the discharge process. Our interventions, as described below, targeted drivers under the locus of control of the ED team.

### The Sedation Board

Our main intervention was to facilitate the organization of sedated reductions by the design, development, and utilization of a centralized sedation whiteboard to list pending sedations, personnel involved, and the status of necessary preparations. In a series of multidisciplinary meetings, we designed the sedation board with the goal of addressing issues of provider and patient readiness to sedate. The sedation board is depicted in Supplemental Digital Content, Figure 1, available at http://links.lww.com/PQ9/A67.

### The Readiness Checklist

We created a sedation “readiness” checklist containing responsibilities of each provider (Supplemental Digital Content, Figure 2, available at http://links.lww.com/PQ9/A68). Copies of the checklist were displayed next to the central sedation communication board and in the designated orthopedic rooms for provider reference and to inform families of the steps involved in the sedation process.

### Postsedation Questionnaire

To facilitate rapid-cycle interventions to improve sedation efficiency, providers completed a postsedation questionnaire regarding possible factors contributing to the prolonged ED LOS. We examined the questionnaire monthly, and we incorporated suggestions into future Plan–Do–Study–Act (PDSA) cycles.

### Family Education

To streamline the consent and discharge processes, we designed family education materials for distribution and discussion with the family before the sedation. These included a “Frequently Asked Questions” (FAQ) document about ketamine and cast care instructions.

All necessary forms (eg, consent forms, Ketamine FAQ, cast care instructions, and postsedation questionnaires) were placed in folders next to the centralized sedation communication board to allow for easy access.

### Promotional Activities

Promotional strategies bolstered the launch of the project and each subsequent Plan–Do–Study–Act cycle. These included new fellow orientation, rotating orthopedic resident orientations, email blasts, “meme-style”-printed advertisements, sharing of baseline and quarterly data updates, ice cream socials, and a raffle.

### Study of the Interventions

We queried our institution’s data warehouse for all patients with *International Classification of Disease*, Ninth or Tenth Revision, diagnosis code for a long bone fracture, a procedure code for procedural sedation, and record of administration of intravenous ketamine from September 2013 through September 2015 to generate baseline data on ED LOS. In addition, these data were captured automatically on a weekly basis for 12 months after the start of interventions and reviewed by project leaders for ongoing measurement and analysis. We gathered an additional 12 months of data to measure sustainability.

### Communication Assessment

To assess baseline interdisciplinary communication before any intervention, we distributed an anonymous online survey to stakeholders in August 2015 to assess barriers to improving sedation LOS. The survey was developed iteratively by project investigators and piloted by a subgroup of stakeholders to ensure construct validity. We emailed the survey to ED providers (physicians, nurses, and clinical assistants). Responders identified their role on the sedation team, described perceived barriers to sedation efficiency, and reported on their perceived overall communication around sedations in the ED on a 5-point Likert Scale. This survey was redistributed to key stakeholders twice after the start of the project (at 4 and 9 months) to assess the interval impact of the intervention on interdisciplinary communication.

### Primary Outcome

The primary outcome measure was the total ED LOS from triage to discharge for patients undergoing ketamine sedation for long bone fractures during the study period. The secondary outcome measure was the change in interdisciplinary communication questionnaire scores.

### Process Measures

We stratified ED LOS into 2 segments of ED care (patient arrival to sedation-start and sedation-end to patient discharge) to determine if the interventions had a differential effect on these components of overall LOS. We also measured the postsedation questionnaire completion percentage.

### Balancing Measures

Balancing measures included LOS for all ED patients and LOS for patients requiring facial laceration repair due to concern that focus on sedated fracture reductions might adversely affect the efficiency of facial laceration repairs, which are also usually performed by fellows or attendings.

### Analysis

We used a pre–post cohort design and assessed differences in outcome, balancing, and process measures over time utilizing statistical process control methodology and conventional guidelines to determine special cause variation centerline shifts.^22^ We performed a log transformation of the highly skewed LOS data, calculated 3-sigma control limits, and then reversed the transformation to show the Y-axis in minutes.^23^ Instances of special cause variation were investigated to determine the root causes.

To assess for differences in patient characteristics, we compared encounters in the preintervention and postintervention periods using the Wilcoxon rank sum test for continuous variables and the Fisher’s exact text for the categorical data. Demographic features that differed significantly between intervention phases could potentially account for changes in the LOS. To address this, we performed a confounder analysis using linear regression with the LOS as the outcome. The model predictors included the intervention phase and all demographics that differed significantly (*P* < 0.05) between the intervention phases.

## RESULTS

There were 1,116 sedations for long bone fracture reduction in the preintervention period (September 2013 to September 2015) and 1,133 in the postintervention period (October 2015 to September 2017). There were no differences in demographics, ED visit characteristics, or injury patterns among patients presenting preintervention and postintervention, except arrival mode, which demonstrated an increase in patients arriving via ambulance from home or as walk-ins and a decrease in those arriving via interfacility transfer (Table 1).

**Table 1.**
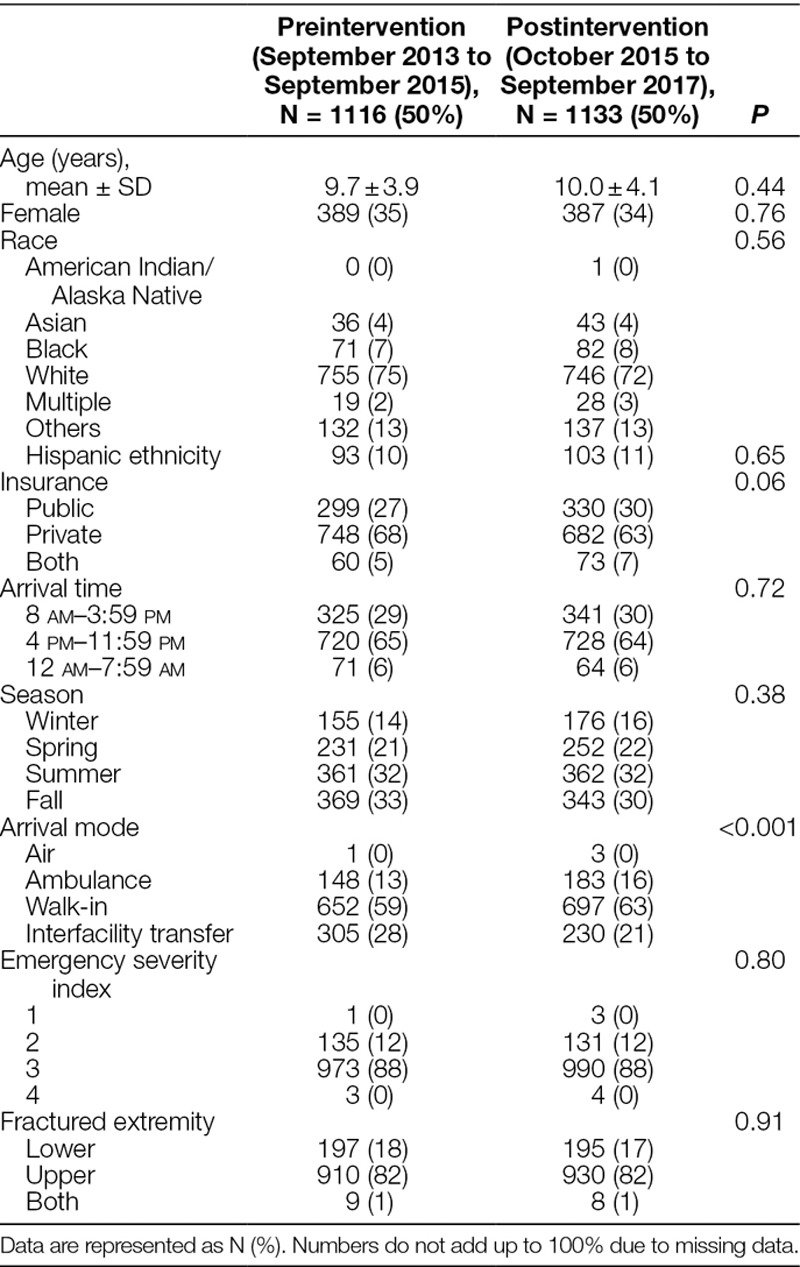
Demographics of Patients before and after Initiation of the Quality Improvement Initiative

### Primary Outcome

**Fig. 3.**
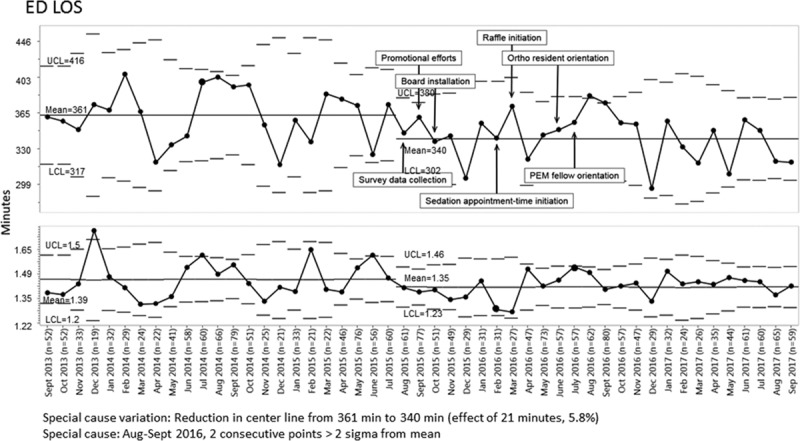
X-bar-S control chart displaying mean emergency department length of stay in minutes—transformed data.

Mean ED LOS decreased by 5.8% from 361 to 340 minutes for ED patients with long bone fracture after implementation of the QI initiative (Fig. 3). We shifted the center line in August 2015, the first of 7 consecutive data points below the prior mean. We noted special cause variation in August 2016 and in September 2016.

Despite an increase in patients arriving either by ambulance from home or as walk-ins after the introduction of the sedation board, LOS still decreased between intervention phases after adjustment for arrival mode (−31 minutes, 95% CI, −44 to −18).

### Process Measures

**Fig. 4.**
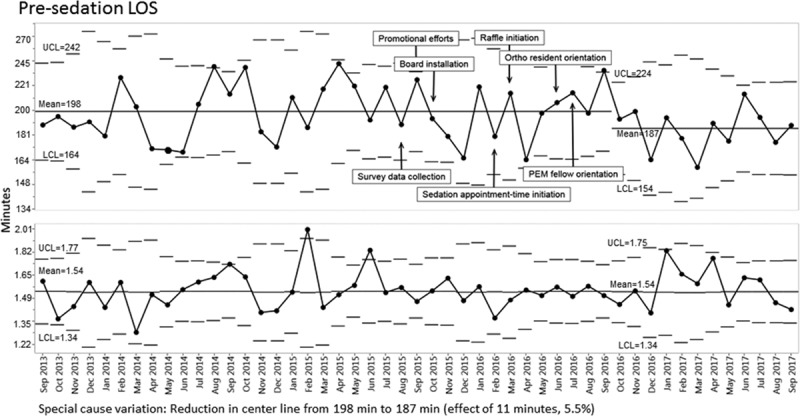
X-bar-S control chart displaying presedation (patient arrival to sedation start) mean emergency department length of stay in minutes—transformed data.

**Fig. 5.**
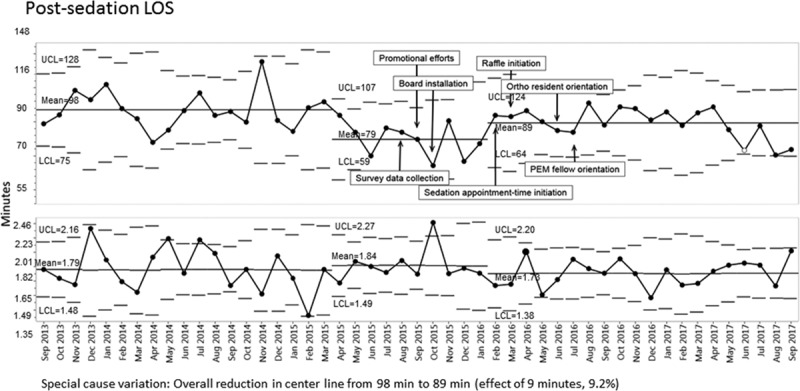
X-bar-S control chart displaying postsedation (sedation end to patient discharge) mean emergency department length of stay in minutes—transformed data.

Time from patient arrival to sedation start declined from 198 to 187 minutes, although the centerline change did not occur until October 2016, 1 year after the introduction of the sedation board (Fig. 4). Time from sedation end to patient discharge also decreased during the study period (Fig. 5).

### Communication

One hundred eight providers completed the preimplementation communication survey, with 58 and 64 completing the survey at 4 and 9 months, respectively. Physicians accounted for the highest survey response rate at each interval (50%, 65.5%, and 67.2% on the preintervention, 4-month postintervention, and 9-month postintervention surveys, respectively). This was followed by nurses (36.1%, 22.4%, and 21.9%) and then clinical assistants (13.9%, 12.1%, and 10.9%). A total of 42 respondents filled out all 3 surveys, whereas 24 respondents filled out 2 surveys and 55 responded to only 1 survey. The proportion of respondents reporting being somewhat or strongly satisfied with communication increased from 68% at baseline to 86% at 4 months (*P* = 0.02) and 92% at 9 months (*P* < 0.001 versus baseline).

The ED provider completed the postsedation questionnaire in 110 of 594 (19%) of sedations in the first 12 months of the intervention period. The forms identified orthopedist, ED physician, and nurse availability as the top 3 perceived contributors to sedation delay throughout the intervention period.

### Balancing Measures

We observed no change in the balancing measure of ED LOS for all ED patients (SDC, Figure 3, available at http://links.lww.com/PQ9/A69) or those patients requiring facial laceration repair (SDC, Figure 4, available at http://links.lww.com/PQ9/A70).

## DISCUSSION

### Summary

A QI initiative collaboratively designed and implemented by pediatric emergency medicine fellows was successful in reducing ED LOS for children undergoing sedated long bone fracture reductions by 5.8% and demonstrated the sustainability of these efforts.

These small but meaningful impacts on LOS along with improvement in provider-reported perception of interdisciplinary communication around sedations represent a better experience for both patients and providers around this resource and time-intensive task.

### Interpretation

There was a 21-minute (5.8%) average reduction in mean ED LOS for our target population. This reduction represents a real difference for patients (who are often in pain and not allowed to eat) and, throughout more than 600 annual sedations for long-bone fracture reduction, adds up to a large impact on overall ED flow. Specifically, any reduction in LOS for these patients can result in shorter stays for other orthopedic patients awaiting reduction and for other patients in the ED or on inpatient floors who might be awaiting these providers.

Notably, although we officially introduced the sedation board in our ED in October 2015, the shift in mean LOS began 2 months earlier in August 2015. We believe that this is likely because in preparing for the introduction of the sedation board, we brought attention to the sedation process and engaged multidisciplinary providers in generating ideas to improve its efficiency. As a result of this attention, efficiency began to improve before the implementation of formal process changes.

Although there was a difference in the arrival mode between the preintervention and postintervention periods, we performed a multivariate linear regression that demonstrated that arrival mode was not associated with LOS, further suggesting that it did not confound the association between intervention phase and LOS.

The initiative did not meet its aim of a 15% reduction in mean LOS in 12 months. This outcome is likely because while we addressed the aspects of the process under control of the sedating physician, we were unable to design interventions addressing other key drivers of ED LOS (eg, number of available orthopedists, ED nurses, and rooms equipped with fluoroscopy).

Of note, the time from ED arrival to sedation start, which should be most affected by the use of the sedation board, did not decrease until 1 year after we implemented the sedation board. This observation may represent a delayed effect of the board, lower statistical power for this particular time segment as opposed to ED LOS as a whole, or the fact that other interventions (such as discharge checklists and family ketamine education) also contributed substantially to the reduction in ED LOS independently of the sedation board. We detected special cause variation in August 2016 and September 2016 with the monthly mean LOS above the upper control limit. An analysis comparing August 2016 to the other intervention months revealed 4 outliers with an ED LOS greater than 700 minutes, which was otherwise rare in our cohort. This month also included the only instance in the study period in which any patients had ED LOS >900 minutes. We feel that the special cause variation seen in August and September 2016 is primarily due to the increased volume of patients requiring sedated fracture reductions, leading to care delays. In August 2016, there was a high monthly census of patients needing sedated fracture reductions (n = 62), and at the same time, evening patient arrivals increased to 5%, suggesting that many of these patients arrived during the busiest time in the ED. Similarly, September 2016 saw the largest number of patients requiring sedated long bone fracture reductions in the entire study period (n = 80). Orthopedist caseload and ED provider availability were consistently noted on our questionnaire as a top contributor to delays in sedation, and both of these are adversely affected in times of high patient volume.

Importantly, we demonstrated an improvement in perceived multidisciplinary communication around procedural sedations within our ED. Improved communication was likely the primary mechanism through which we were able to improve ED LOS. Improved communication has value in and of itself as well in leading to improved patient safety and as such is congruent with the national agenda set forth by The Joint Commission.^24^

### Limitations

Our study had several limitations. First, it is possible that our data capture model missed some sedations that occurred during the intervention period. To address this, we designed and refined the data capture mechanism to minimize such omissions, which were unlikely to have biased the results in either direction. Second, we focused our interventions on the aspects of the sedation process under the control of the ED staff as opposed to targeting other potentially impactful factors, such as ED orthopedic staffing models or dedicated sedation nurses. In particular, we were unable to predict or affect the orthopedic provider’s availability based on their potential simultaneous involvement in operating room cases, inpatient orthopedic floor patient needs, or the number of concurrent orthopedic consultations in the ED. Third, a possible secular trend of improved interdisciplinary communication may have contributed to our demonstrated improvements in perceived communication, rather than the intervention itself. Fourth, our communication survey data are limited by biases inherent to survey methodology, namely the use of a convenience sample subject to recall bias. Also, the survey was distributed at 4 and 9 months, but not at the conclusion of the study, thereby potentially missing changes in the perceived improvement in communication. The survey response rates at 4 and 9 months were also notably decreased in comparison to our initial survey, thereby limiting our assessment of improvement. Finally, we set our primary intervention, and thereby the start date of our postintervention period, as the initiation and hanging of our sedation board (October 2015). However, there were interventions ongoing from August 2015 to July 2016, and therefore, these definitions of “preintervention” and “postintervention” may not be precise. We believe, however, that most of these interventions were in support of the primary intervention, the sedation board, and as such chose that to define the periods of the study.

Despite these limitations, we believe this study to be important as we postulate that improvements in ED LOS and interdisciplinary communication will lead to downstream improvements in patient safety, patient perceptions of provider teamwork, appropriate distribution of staffing, and most importantly, the patient’s positive overall experience under our care.

### Conclusions

A collaborative, fellow-driven QI project was successful in sustainably decreasing LOS for patients undergoing sedated long bone fracture reductions in a pediatric ED and led to improvements in perceived interdisciplinary staff communication. These improvements likely had beneficial effects on overall ED operations and the patient experience.

## DISCLOSURE

The authors have no financial interest to declare in relation to the content of this article.

## Supplementary Material
