# Supplementary material for: Improving Efficiency and Communication around Sedated Fracture Reductions in a Pediatric Emergency Department
Source: Pediatr Qual Saf. 2019 Feb 13;4(1):e135. doi: 10.1097/pq9.0000000000000135 (PMC6426494; doi:10.1097/pq9.0000000000000135)
Supplement: Supplementary file 1 [file pqs-4-e135-s001.docx]

**Improving Efficiency and Communication Around Sedated Fracture Reductions in a Pediatric Emergency Department: A Quality Improvement Initiative**

**First Author:** Niloufar Paydar-Darian

**SDC, Figure 1:** Sedation Communication Board

**
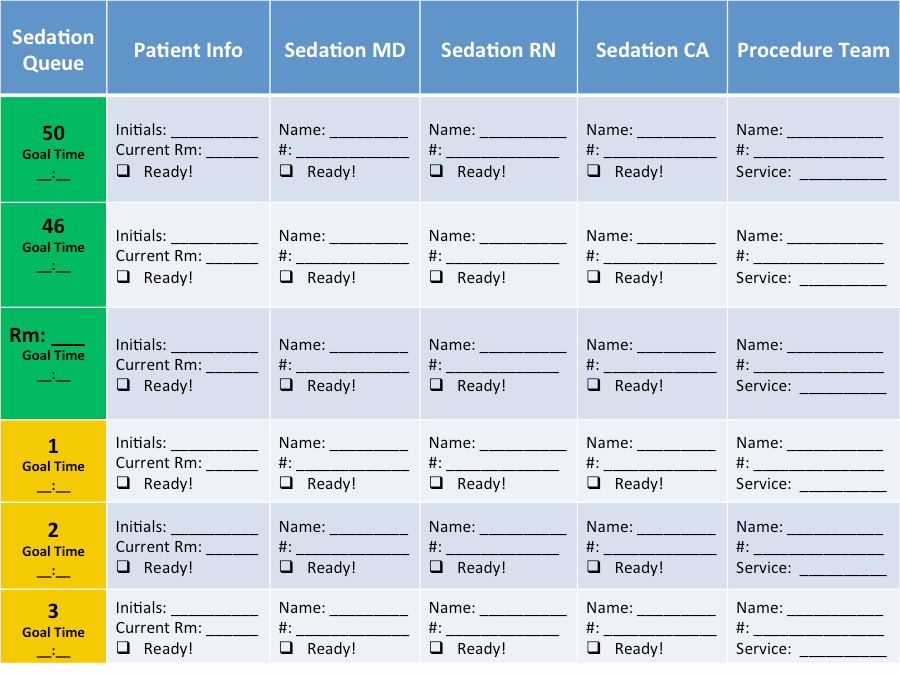
**

The final sedation communication board consists of mobile magnetic rows on a white board. Each row includes the patient’s current location, initials, name and contact information for staff performing the sedation (e.g. sedation MD, procedure MD, RN, and CA), the sedation room assignment, and the goal start time. Rows starting with a green-colored box reflect patients in sedation-appropriate rooms. Rows starting with a yellow-colored box reflect those in queue for sedation and awaiting transfer to sedation-appropriate room. Check boxes within the patient’s row allow providers to notify their colleagues that necessary pre-sedation tasks are complete. Patient magnets can be manipulated to designate the order of sedations in the ED. The board was placed in a central location, easily visible from the Charge Nurse station, in order to facilitate communication and use by all members of the ED sedation team.
