# Supplementary material for: Improving Efficiency and Communication around Sedated Fracture Reductions in a Pediatric Emergency Department
Source: Pediatr Qual Saf. 2019 Feb 13;4(1):e135. doi: 10.1097/pq9.0000000000000135 (PMC6426494; doi:10.1097/pq9.0000000000000135)

**Improving Efficiency and Communication Around Sedated Fracture Reductions in a Pediatric Emergency Department: A Quality Improvement Initiative**

**First Author:** Niloufar Paydar-Darian

**SDC, Figure 2:** Sedation Readiness Checklist


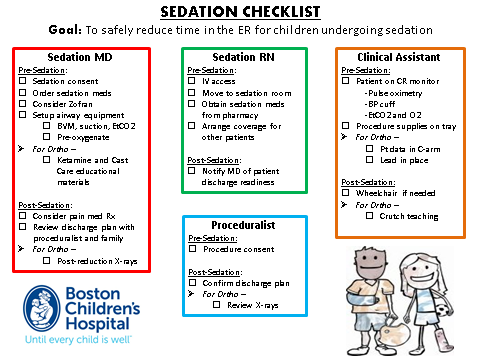

Supplement: Supplementary file 2 [file pqs-4-e135-s002.docx]
