# Supplementary figures and images for: Improving Efficiency and Communication around Sedated Fracture Reductions in a Pediatric Emergency Department
Source: Pediatr Qual Saf. 2019 Feb 13;4(1):e135. doi: 10.1097/pq9.0000000000000135 (PMC6426494; doi:10.1097/pq9.0000000000000135)

SDC, Figure 3: Median Length of Stay for all Emergency Department Patients

Run chart

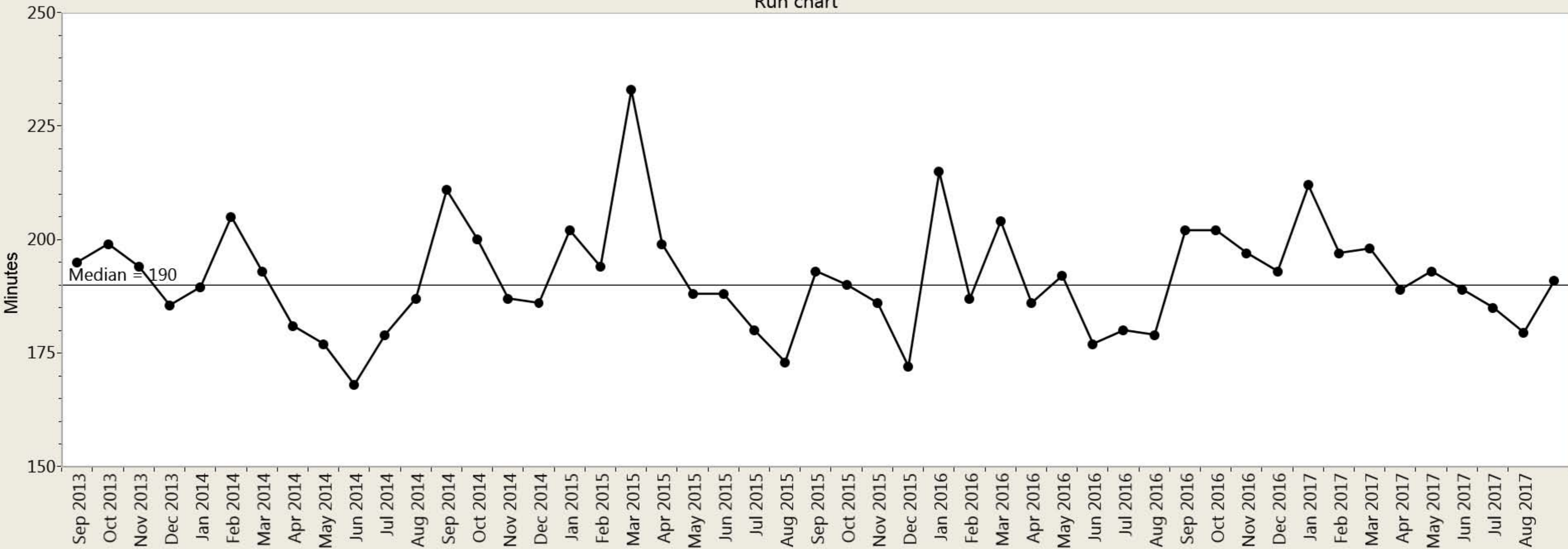

Supplement: Supplementary file 3 [file pqs-4-e135-s003.pdf]

SDC, Figure 4: Median Length of Stay for Facial Laceration Patients in the Emergency Department

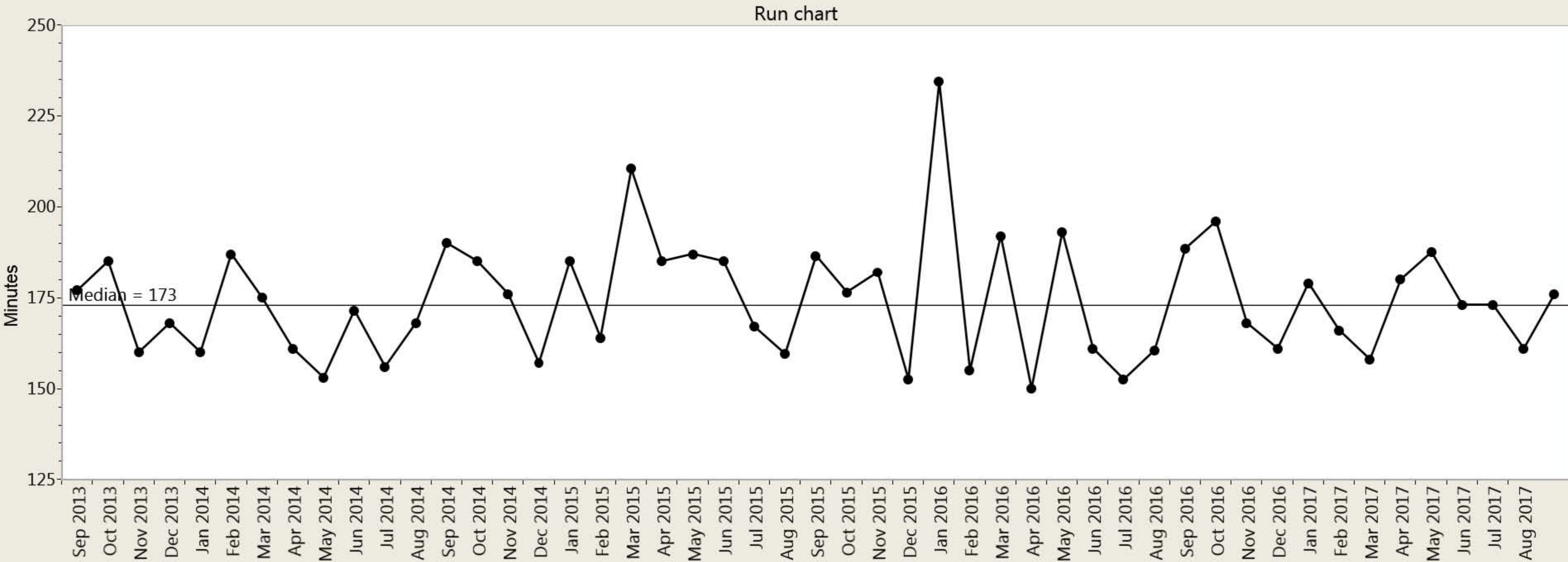

Supplement: Supplementary file 4 [file pqs-4-e135-s004.pdf]
